# Does synbiotic supplementation affect the quality of life in children with cystic fibrosis? A pilot randomized controlled clinical trial

**DOI:** 10.1186/s40795-020-00373-4

**Published:** 2020-10-15

**Authors:** Nemat Bilan, Effat Marefat, Leila Nikniaz, Mahdieh Abbasalizad Farhangi, Zeinab Nikniaz

**Affiliations:** 1 Pediatric Health Research Center, Tabriz University of Medical Sciences, Tabriz, Iran; 2 Tabriz Health Services Management Research Center, Tabriz University of Medical Sciences, Tabriz, Iran; 3 Drug applied research center, Tabriz University of medical sciences, Tabriz, Iran; 4 Liver and Gastrointestinal Diseases Research Center, Tabriz University of Medical Sciences, Tabriz, Iran

**Keywords:** Quality of life, Cystic fibrosis, Synbiotic, Children

## Abstract

**Background:**

There is no clinical trial that assesses the effect synbiotic supplementation on HRQOL in CF children. Considering the importance of HRQOL as an essential primary outcome and determinant of therapeutic benefit in chronic diseases like cystic fibrosis, the present clinical trial aimed to determine the efficacy of synbiotic supplementation on HRQOL in children with CF.

**Methods:**

In the present double-blind randomized clinical trial, 40 CF children were randomly allocated to the two groups. The intervention group was supplemented with synbiotics supplements and the patients in the placebo group received maltodextrin for 6 months. Demographic data and information about antibiotic use were recorded using the questionnaire. The health-related quality of life was assessed using the Persian version of quality of life inventory questionnaires. Paired t-test and ANCOVA were used for statistical analysis.

**Results:**

Totally, 36 participants completed the trial. The mean score of HRQOL was 76.34 ± 17.33. There were no significant differences between synbiotic and placebo groups regarding baseline demographic and quality of life characteristics. Compared with baseline values, the mean total score and subscores of quality of life did not change significantly after synbiotic and placebo supplementation (*p* > 0.05). Moreover, the results of ANCOVA showed that there were no significant differences between the two groups regarding the post-trial value of HRQOL total score and subscores.

**Conclusion:**

According to results, six-month supplementation with synbiotic did not have a significant effect on the HRQOL in children with CF. However, further studies with larger sample sizes and using more disease-specific questionnaires are needed for a more precise conclusion.

The protocol of the study was registered at Iranian registry clinical trials (Registration code: IRCT2017011732004N1; Registration date: 2017-02-14).

## Background

Cystic fibrosis (CF) is a multi-organ and life-limiting disease [1] that is associated with respiratory infections and gastrointestinal inflammation with a possible association with intestinal dysbiosis [2, 3]. According to earlier reports, treatment interventions, special dietary regimen, and also intestinal dysfunction usually resulted in gastrointestinal dysbiosis in CF children that may contribute to different complications [4]. Considering the effect of probiotic bacteria on pathogen bacteria, different studies were conducted to assess the effect of probiotics supplementation in CF patients and some of them reported promising results [5–9]. However, only limited data is available about the effect of this supplement on health-related quality of life (HRQOL) in CF children. The only previous study that examined the effect of one-month supplementation of probiotics (2 × 10^9^ CFU/d) on quality of life in CF children showed that there was no significant difference between intervention and placebo groups regarding children report of HRQOL. However, the parents’ report indicated significant improvement in the physical and total score of quality of life [10]. Supplementation duration in this study was limited and it seems that a study with longer treatment duration would be of value. On the other hand, previous studies showed that synbiotic supplements (have both prebiotic and probiotic properties) may have a synergistic effect on the intestinal microbiota [11, 12]. However, there is no clinical trial that assesses the effect synbiotic supplementation on HRQOL in CF children. In our previous publication on the same sample, we showed that although the mean hospitalization period and number of pulmonary exacerbation were lower in synbiotic group compared with placebo group, we did not reach statistically significant difference. This may be due to the short follow-up period [13].

Considering the importance of HRQOL as an essential primary outcome and determinant of therapeutic benefit in chronic diseases like cystic fibrosis, the present clinical trial aimed to determine the efficacy of synbiotic supplementation on HRQOL in children with CF.

## Methods

In the present double-blind randomized placebo-controlled clinical trial, the CF patients were selected from Tabriz Children’s Hospital affiliated with Tabriz University of medical sciences. The diagnosis of CF was confirmed by a pediatrician according to clinical signs and two sweat chloride test (> 60 mmol/L). The children were included if they aged 5–12 years with Tiffeneau-Pinelli index> 40% and the absence of recent acute exacerbation. The exclusion criteria were: having other related diseases such as liver or endocrine diseases, having ventilator-dependent respiratory failure, or had regular use of probiotics and probiotic fortified food were excluded from the study. Forty children with CF met inclusion/exclusion criteria and entered the study.

Written informed consent was obtained from all children’s parents. This study was approved by The Ethics Committee of Tabriz University of Medical Sciences) IR.TBZMED.REC.1395.919). The protocol was registered at Iranian registry clinical trials (IRCT2017011732004N1).

### Experimental design

Forty patients were randomly divided into intervention and placebo groups according to their age and sex. The detailed methods of randomization, blinding and deciding about patients’ adherence to study protocol were described in our previous publication [13]. The sample size was calculated using the G-power software and based on the result of the previous study [10] and considering the changes in PedsQL total score (changes in PedsQL total score in the synbiotic group: 2.37 ± 1.17, and placebo group: 1.4 ± 0.68), 90% power, two-sided 5% significance and 20% drop-out rate. This necessitates the sample size of 20 participants in each group.

The patients in the intervention and placebo groups were instructed to consume two synbiotic or maltodextrin capsules per day for 6 months. The synbiotic supplement was purchased from Zist Takhmir company and its content was reported in our previous publication [13].

### Outcome measures

In this clinical trial, different outcomes including anthropometric measurements, hospitalization duration, antibiotic use, clinical outcomes, and quality of life were studied. All outcomes except the quality of life outcomes were reported in our previous study [13]. In the present study, we reported the effect of synbiotic supplementation on HRQOL in children with CF.

The health-related quality of life was assessed using the Persian version of quality of life inventory questionnaires (PedsQL) [14]. The validity of the Iranian version of this questionnaire was investigated previously [14]. The questionnaires had 23 items on a five-point Likert scale ranging from 0 (never a problem) to 4 (almost always a problem). According to the questionnaire instruction [15], the scores were transformed on a scale from 0 (score 4) to 100 (score 0) and the total score and dimensions scores were calculated by summing of the items over the number of items answered. The questionnaire includes the following dimension scores: physical (8 questions), emotional (5 questions), social (5 questions), and school functioning (5 questions). The final HRQoL total score and dimension scores were computed out of 100. A change of 4.4 in the PedsQL summary score was considered as the minimal clinically important difference (MCID) [16]. The questionnaire was completed before and 6 months after supplementation.

### Statistical analysis

All analyses were conducted using SPSS 22.0 and based on intention to treat (ITT) analysis. The Kolmogorov-Smirnov test was used for checking the normality. The within-group comparisons were performed by paired sample t-test. The between-group analysis was done using independent sample t-test and chi-square tests. One way ANCOVA was used to compare the quality of life score after intervention by adjusting to the baseline values. The significance level was considered *P*-value ≤0.05.

## Results

**Fig. 1:**
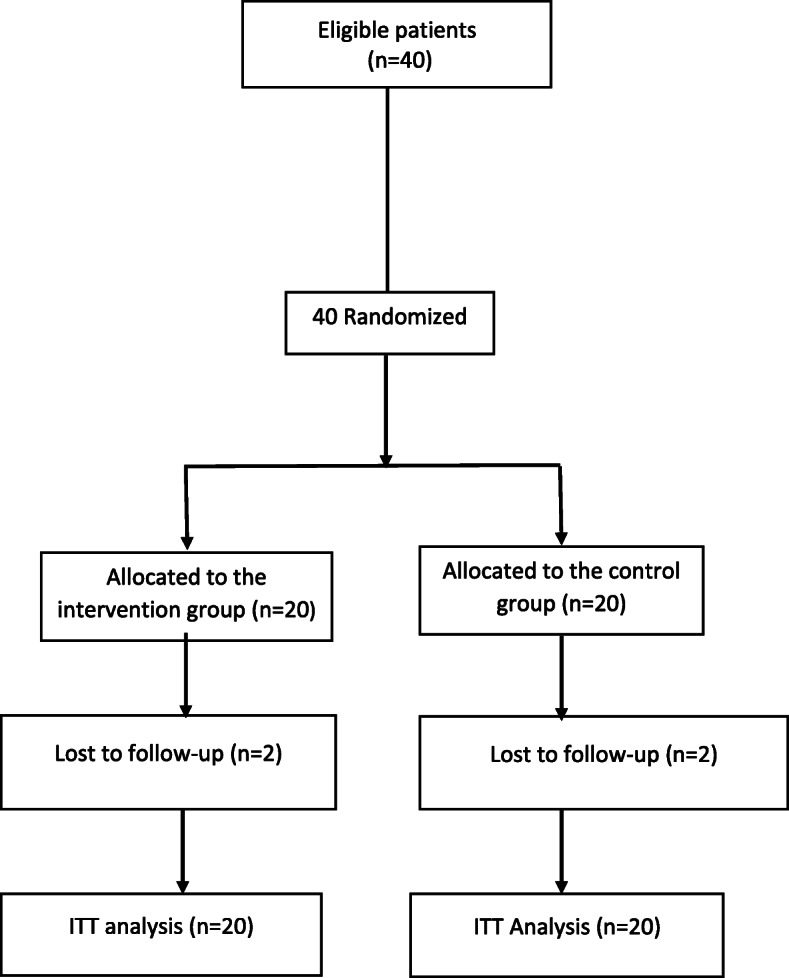
Flow chart of patients’ recruitment and analysis

Figure 1 shows the patients` recruitment and analysis diagram. According to the figure, thirty-six participants completed the trial. The patient’s mean age was 8.72 ± 3.23 years and 52.5% of them were male. The mean Forced expiratory value in 1 s (FEV_1_) (%) was 81.75 ± 27.51 and 80.29 ± 22.84 in synbiotic and placebo groups respectively. The mean score of quality of life was 76.34 ± 17.33. There were no significant differences between synbiotic and placebo groups regarding baseline demographic and quality of life characteristics (Table 1).

**Table 1:** Baseline characteristics of participants

Variables	Synbiotic (***n*** = 20)	Placebo (n = 20)	***p***-value*
**Age (years)**	8.29 ± 2.11	9.25 ± 3.99	0.35
**Sex (M/F)**	9/11	12/8	0.26**
**FEV1 (%)**	81.75 ± 27.51	80.29 ± 22.84	0.68
**Quality of life (total score)**	72.76 ± 17.96	79.92 ± 16.42	0.23
**Physical score**	67.64 ± 22.93	73.71 ± 19.48	0.41
**Emotional score**	66.47 ± 22.62	79.41 ± 22.63	0.10
**Social score**	79.41 ± 22.21	82.05 ± 10.61	0.66
**School performance score**	80.58 ± 19.43	88.23 ± 18.70	0.25

*M* Male, *F* Female, *FEV* Forced Expiratory Volume

*p-value of independent t-test

**p-value of chi-square

Table 2 presents the mean total score and subscores of quality of life in synbiotic and placebo groups. As can be seen, compared with baseline values, the mean total score and subscores of quality of life did not change significantly after probiotic and placebo supplementation (*p* > 0.05). Moreover, the results of ANCOVA showed that there were no significant differences between the two groups regarding the post-trial value of quality of life total score and subscores. Moreover, the changes were not clinically meaningful.

**Table 2:** Comparison of the quality of life score and subscores between two groups

Variables	Synbiotic (***n*** = 20)	Placebo (***n*** = 20)	***p***-value
**Quality of life (total score)**			
**Before**	72.76 ± 17.96	79.92 ± 16.42	0.23^**^
**After**	73.52 ± 16.74	79.95 ± 15.57	0.95^¥^
*p*-value^*^	0.08	0.69	
**Physical score**			
**Before**	67.64 ± 22.93	73.71 ± 19.48	0.41^**^
**After**	69.30 ± 20.40	74.26 ± 18.37	0.58^¥^
*p*-value^*^	0.07	0.48	
**Emotional score**			
**Before**	66.47 ± 22.62	79.41 ± 22.63	0.10^**^
**After**	66.17 ± 21.39	79.37 ± 21.28	0.74^¥^
*p*-value^*^	0.57	0.61	
**Social score**			
**Before**	79.41 ± 22.21	82.05 ± 10.60	0.66^**^
**After**	80.00 ± 20.76	82.04 ± 9.69	0.75^¥^
*p*-value^*^	0.33	0.91	
**School performance score**			
**Before**	80.58 ± 19.43	88.23 ± 18.70	0.25^**^
**After**	81.17 ± 19.08	89.11 ± 18.64	0.69^¥^
*p*-value^*^	0.33	0.45	

*p-value of paired sample t-test

**p-value of independent sample t-test

¥ *p*-value of ANCOVA after adjusting for baseline values

## Discussion

Considering the association of CF with intestinal dysbiosis, in the present study, we studied the effect of synbiotic supplementation on HRQOL in children with CF. HRQOL is an important and essential primary outcome and determinant of therapeutic benefit in chronic diseases such as cystic fibrosis. Previously, different studies have assessed the effect of probiotic supplementation on other childhood respiratory and gastrointestinal diseases and providing conflicting results. For example, Huang et al. showed that the probiotic supplementation did not significantly affect the QOL in children with asthma [17]. However, Guandalini, et al. showed the promising effect of the probiotic mixture on QOL in children with irritable bowel syndrome [18].

In the present study, we showed that compared with placebo, synbiotic supplementation did not have a significant effect on the quality of life in these patients. In addition, considering the MCID of 4.5, the differences were not clinically important. To the best of our knowledge, only limited data is available regarding the effect of probiotic supplementation on quality of life in these patients. In line with the results of the present study, Jaffari et al. also showed no significant difference between intervention and placebo groups regarding children’s reports of health-related quality of life. However, the parents’ report indicated significant improvement in the physical and total score of quality of life [10]. In the present study, we did not assess the parents’ report of child HRQOL. The observed effect of probiotic supplementation on parents’ reports of HRQOL in the mentioned study may be attributed to the differences in disease severity. Although Jaffari et al., did not report the FEV1, none of their patients received antibiotics during the intervention period. However, in the present study, 35.3 and 55% of patients in the intervention and placebo groups (*p* = 0.32) respectively received antibiotics during the study [13]; this may be due to a longer duration of supplementation or higher severity of diseases in the present study.

The observed lack of significant effect of synbiotic supplementation on HRQOL in CF children in the present study may be partly due to the lack of its effect on pulmonary exacerbation [13]. Some studies reported the positive effect of probiotic supplementation on pulmonary and intestinal manifestations. So, we postulated that through this mechanism, synbiotic may improve HRQOL in CF children. However, we showed that this supplement did not reduce pulmonary exacerbation [13] and nor did HRQOL in CF patients.

The results of the present study should be interpreted according to the following limitations. We did not use the CF specific quality of life assessment questionnaire. We could not find any Persian-language CF-specific HRQOL questionnaire, however, we used a valid general questionnaire to assess health-related quality of life. We did not have a follow-up period to assess the long term effect of synbiotic supplementation on quality of life. We postulated that a longer follow-up period may positively affect the clinical symptoms and consequently the quality of life in the synbiotic group.

## Conclusion

Briefly, According to the results, six-month supplementation with synbiotic did not have a significant effect on the quality of life total score and subscores in children with CF. To the best of our knowledge, this is the first study that assesses the effect of synbiotic in these patients and the data regarding the effect probiotic supplements on quality of life in these patients are also scarce. So, further studies with a longer follow-up period and using more disease-specific questionnaires are needed for a more precise conclusion.

## Data Availability

The datasets generated and/or analysed during the current study are not publicly available due to due institution’s policy but are available from the corresponding author on reasonable request.

## Abbreviations

ANCOVA: One-way Analysis of Covariance (ANCOVA)
CF: Cystic fibrosis
FEV1: Forced expiratory value in one score
HRQOL: Health-related quality of life
